# CPT1α maintains phenotype of tubules via mitochondrial respiration during kidney injury and repair

**DOI:** 10.1038/s41419-021-04085-w

**Published:** 2021-08-14

**Authors:** Qi Yuan, Yunhui Lv, Hao Ding, Qingqing Ke, Caifeng Shi, Jing Luo, Lei Jiang, Junwei Yang, Yang Zhou

**Affiliations:** 1 Center for Kidney Disease, Second Affiliated Hospital of Nanjing Medical University, Nanjing, China; 2 Department of Nephrology, The Affiliated Suzhou Hospital of Nanjing Medical University, Suzhou Municipal Hospital, Gusu School, Nanjing Medical University, Suzhou, China

**Keywords:** Mechanisms of disease, Chronic kidney disease, Pathogenesis

## Abstract

Impaired energy metabolism in proximal tubular epithelial cells (PTECs) is strongly associated with various kidney diseases. Here, we characterized proximal tubular phenotype alternations during kidney injury and repair in a mouse model of folic acid nephropathy, in parallel, identified carnitine palmitoyltransferase 1α (CPT1α) as an energy stress response accompanied by renal tubular dedifferentiation. Genetic ablation of *Cpt1α* aggravated the tubular injury and interstitial fibrosis and hampered kidney repair indicate that CPT1α is vital for the preservation and recovery of tubular phenotype. Our data showed that the lipid accumulation and mitochondrial mass reduction induced by folic acid were persistent and became progressively more severe in PTECs without CPT1α. Interference of CPT1α reduced capacities of mitochondrial respiration and ATP production in PTECs, and further sensitized cells to folic acid-induced phenotypic changes. On the contrary, overexpression of CPT1α protected mitochondrial respiration and prevented against folic acid-induced tubular cell damage. These findings link CPT1α to intrinsic mechanisms regulating the mitochondrial respiration and phenotype of kidney tubules that may contribute to renal pathology during injury and repair.

## Introduction

The kidney requires a large amount of ATP to remove waste and reabsorb nutrients, to modulate the balance of electrolytes, fluid, and acid-base homeostasis [1]. Energy metabolism in kidney proximal tubular epithelial cells (PTECs) is particularly unique because ATP production here primarily depends on oxidative phosphorylation (OXPHOS) of fatty acid in mitochondria which is more efficient and safe in energy generation by oxidation of glucose [2, 3]. Evidence has accumulated to show that PTECs are not uninvolved in the process of kidney injury. They undergo phenotypic changes, acquire mesenchymal functions, and hence contribute to the synthesis of extracellular matrix proteins [4, 5]. The high-energy requirement and the significance of energy metabolism in tubular cells have been appreciated for many years; however, a strong link between impaired metabolic homeostasis and renal injury has only emerged recently [6].

After kidney injury, the PTECs die or undergo dedifferentiation and proliferation to repair [7]. Redifferentiation of the reconstituted epithelium is the major pathway towards the recovery of normal kidney structure and function. However, this is merely an ideal condition because the redifferentiation of proliferative PTECs varies [8]. Those PTECs failed to redifferentiate become atrophy [9] and trigger tubulointerstitial fibrosis [10]. Although the mechanism by which the PTECs become redifferentiation or atrophy is unknown, the energy metabolism is probably involved. Metabolism alternations occur immediately after injury, including diminished fatty acid oxidation (FAO) [6], activated glycolysis [11], increased lactate release [12], and elevated pyruvate kinase in the kidney [13]. These physiologic changes in regenerating epithelium may become pathologic if persistent. The previous study has suggested that tubules with a glycolytic shift and mitochondrial pathology in the early stage of regeneration after kidney injury probably fail to redifferentiate and progress to atrophy [9].

Transcriptome analysis demonstrated a markedly decreased expression of key enzymes and regulators of FAO in kidney diseases [6]. Recently, through unbiased cell trajectory analyses, it revealed that differentiation of PTECs was altered in kidney disease, which was strongest and most reproducible associated with OXPHOS and FAO in tubules [14]. The first and rate-limiting component of FAO system is carnitine palmitoyltransferase 1 (CPT1). Several studies indicated that CPT1 protects against kidney disease [6, 15–18].

On the basis of this, we hypothesized that CPT1α is involved in the mechanism of the maintenance of PTECs phenotype and that its dysregulation is related to the development of tubular damage, poor repair, and subsequent fibrosis. To address these hypotheses, we evaluated renal lipid and the tubular phenotype of inducible PTEC-specific *Cpt1α*-deficient mice exposed to a folic acid-induced mouse model of kidney injury and repair.

## Material and methods

### Animal model

Male C57BL/6J mice purchased from Shanghai experimental animal center were housed in the animal facilities at Nanjing Medical University and were treated humanely according to guidelines of the Institutional Animal Use and Care Committee with free access to water and food. A conditional system was used to knockout CPT1α in the renal tubule of adult mice. Three transgenic mouse lines were cross-bred: Pax8-reverse tetracycline-dependent transactivator (Pax8-rtTA) mice (Jackson lab, stock No: 007176), tetO mice (Jackson lab, stock No: 006234) and CPT1α^flox/flox^ mice, which were in-housed generated, with exon 3 of the mouse *Cpt1α* gene floxed. Tail DNA from all mice was genotyped by PCR analysis. Primers used for CPT1α^flox/flox^ genotyping were as follows: Primer F 5′-GCA GCC CAG CTG ATG ACC TGA G-3′; Primer wild-type R 5′-CCT CTG CCA CTC TTA GCC TAG TC-3′; Primer neo R 5′-TGC TAA AGC GCA TGC TCC AGA C-3′. Doxycycline-containing chow was started at 3 weeks of age. Male mice aged 6−8 weeks were randomly assigned into different groups with at least six mice per group: control, 2 weeks, 4 weeks, and 12 weeks after folic acid. Control mice were injected intraperitoneally with NaHCO_3_ (300 mmol/L). Folic acid (F7876, Sigma-Aldrich) was dissolved in NaHCO_3_ (300 mmol/L) and injected intraperitoneally at the dose of 250 mg/kg [6, 19]. No blinding was done.

### Cell culture and treatment

Primary PTECs were cultured from collagenase-digested cortical fragments of mice (about 21 days) kidneys according to a modified method previously described [20, 21]. In brief, after been dissected and collagenase digested, two nylon sieves with the pore sizes of 250 and 80 μm were used to yield proximal tubule fragments, which were then washed, resuspended, and seeded onto collagen-coated permeable PTFE-filter supports and cultured for 48 h in a standard humidified incubator with the medium replaced every 2 days till the organization of a confluent monolayer of cells. At 80% confluence, cells were starved overnight and treated with 10 mmol/L folic acid (FA) [22, 23]. PTECs were transiently transfected with CPT1α siRNA or corresponding negative control (Integrated Biotech Solutions Co., Ltd, Shanghai, China) by lipofectamine RNAiMAX transfection reagent (13778, Invitrogen). CPT1α plasmid (pCPT1α) and control plasmid (pcDNA3) were transfected at 4 μg/ml by lipofectamine 3000 reagent (L3000, Invitrogen). Twenty-four hours later, PTECs were then exposed to FA. The siRNA sequences of CPT1α and negative control were as follows: CPT1α: sense 5′- GGA GGA GGU AAG ACU ACU AUG-3′; anti-sense 5′- UAG UAG UCU UAC CUC CUC CUU-3′. Negative control (N.C.): sense 5′-UUC UCC GAA CGU GUC ACG UTT-3′; anti-sense 5′-ACG UGA CAC GUU CGG AGA ATT-3′.

### Renal function assay

Blood urea nitrogen (BUN) and serum creatinine were measured by QuantiChrom Urea and Creatinine Assay kit (DIUR-500, DICT-500, Hayward, CA), respectively.

### Morphology assay

Kidney samples were fixed in 10% of neutraformalin in 4 °C overnight followed by paraffin-embedded and sectioned (3 μm in thickness) for Masson trichrome or Sirius red staining. Slides were viewed with a Nikon Eclipse 80i microscope (DS-Ri1, Nikon, Shanghai, China). The fibrotic area (%) for each section was calculated using Image-Pro Plus 6.0 software. Ten randomly chosen fields were evaluated for each mouse, and an average score was calculated.

### Transmission electron microscopy

Kidney sections were fixed in glutaraldehyde (3.7%) followed by osmium tetroxide (1%) and were embedded in gelatin (10%) to cut into several blocks (<1 mm^3^). After dehydration and infiltration in increasing concentrations of alcohol and Quetol-812 epoxy resin mixed with propylene oxide, respectively, samples were embedded in fresh Quetol-812 epoxy resin and polymerized. Sections were cut into 100 nm in thickness and post-stained with uranyl acetate (10 min) and lead citrate (5 min) and finally observed in a FEI Tecnai T20 TEM (Thermo Fisher Scientific, Carlsbad, CA, USA), operated at 120 kV. The number of mitochondria per field (*N*_V_, n/μm3) was estimated as previously described [24]. Briefly, mitochondrial profile area density (*N*_A_) was the ratio between the mitochondrial number and proximal tubular area. Mitochondrial volume density (*V*_V_) was the ratio of grid points falling over mitochondria and the total number of points of the grid container in the proximal tubule. *N*_V_ = (1/*β*) (*N*_A_^3/2^/*V*_V_^1/2^), where *β* is calculated using the ratio of the harmonic mean of major and minor axes of mitochondrial sections measured on digital images.

### Mitochondrial DNA copy number determination

Genomic DNA was extracted using the DNeasy Blood & Tissue Kit (69504, Qiagen). The abundance of mitochondrial DNA (mtDNA) was measured using Mouse mtDNA Copy Number Assay Kit (MCN3, Detroit R&D). Relative mtDNA copy number was the ratio of mtDNA to nuclear DNA.

### Western immunoblot analysis

Western blot was performed using a lysate of kidney cortex or cultured PTECs. The primary antibodies used were as follows: anti-CPT1α (ab128568, Abcam), anti-KIM1 (ab47635, Abcam), anti-NGAL (ab63929, Abcam), anti-E-cadherin (610181, BD Company), anti-AQP1 (ab168387, Abcam), anti-vimentin (ab92547, Abcam), anti-fibronectin (F3648, Sigma Aldrich), anti-collagen I (1310-01, Southern Biotech) and anti-Tubulin (T6074, Sigma Aldrich). Western blot was performed three times independently. Quantification was completed by scanning and analyzing the intensity of hybridization signals by using NIH Image program.

### Immunohistochemical staining

Paraffin-embedded kidney sections were applied to immunohistochemical staining. They were stained with CPT1α antibody (ab128568, Abcam), KIM-1 antibody (SAB3500252, Merck), E-cadherin antibody (610181, BD Company), collagen I antibody (1310-01, Southern Biotech) and fibronectin antibody (F3648, Sigma Aldrich) using the Vector Mouse on Mouse (M.O.M.) immunodetection Kit (Vector Laboratories, Burlingame, CA). Isotype control was also performed.

### Immunofluorescent staining

Kidney tissue cryosection (3 μm in thickness) was fixed in paraformaldehyde (4%), permeabilized with Triton X-100 (0.2%), blocked with donkey serum (2%), and then immunostained with antibodies. Similarly, cells were washed, fixed, blocked, and then incubated with specific antibody: E-cadherin antibody (610181, BD Company), anti-AQP1 (AB2219, Millipore), anti-vimentin (sc-6260, Santa), anti-laminin (ab11575 or ab44941, both from Abcam), anti-collagen I (1310–01, Southern Biotech), or anti-fibronectin (F3648, Sigma Aldrich). Secondary antibodies were FITC or TRITC-conjugated. Cell nuclei were visualized by 40, 6-diamidino-2-phenylindole (DAPI) staining. Slides were viewed under a confocal inverted laser microscope (LAM 510 Meta, Zeiss).

### Lipid droplets staining

OCT-embedded kidney tissues were sectioned at 12 μm for oil red O (Sigma-Aldrich, US) and 3 μm for bodipy (D3922, Thermo Fisher, US) staining as previously described [6, 21, 25]. Nuclei were viewed by alum haematoxylin staining. After bodipy staining, slides were immunostained with laminin (L9393, Sigma-Aldrich) and DAPI to visualize the tubule and cell nuclei, respectively. The positive area (%) for each section was analyzed using Image-Pro Plus 6.0 software. At least ten randomly chosen fields were evaluated for each sample, and an average score was calculated.

### Measurement of oxygen consumption rate (OCR)

OCR was measured using a Seahorse Bioscience X24 extracellular flux analyzer (XF24 V7, Seahorse Bioscience). PTECs were seeded in XF24 V7 cell culture microplate at a 1.0 × 10^4^ cells per well. OCR (pmol/min) was assessed at baseline and after the addition of 1 μmol/L of oligomycin, followed by 0.75 μmol/L of carbonyl cyanide 4-(trifluoromethoxy) phenylhydrazone (FCCP), and finally 1 μmol/L of oligomycin and rotenone. Protein concentration was measured for normalization.

### Statistical analysis

Statistical analysis of data was performed using Sigma Stat software (Jandel Scientific Software, San Rafael, CA). Data were expressed as mean ± SD. Comparisons between groups were made using one-way ANOVA, followed by the *t* test. *P* < 0.05 was considered significant.

## Results

### Expression of CPT1α during kidney injury and repair in folic acid nephropathy model

**Fig. 1:**
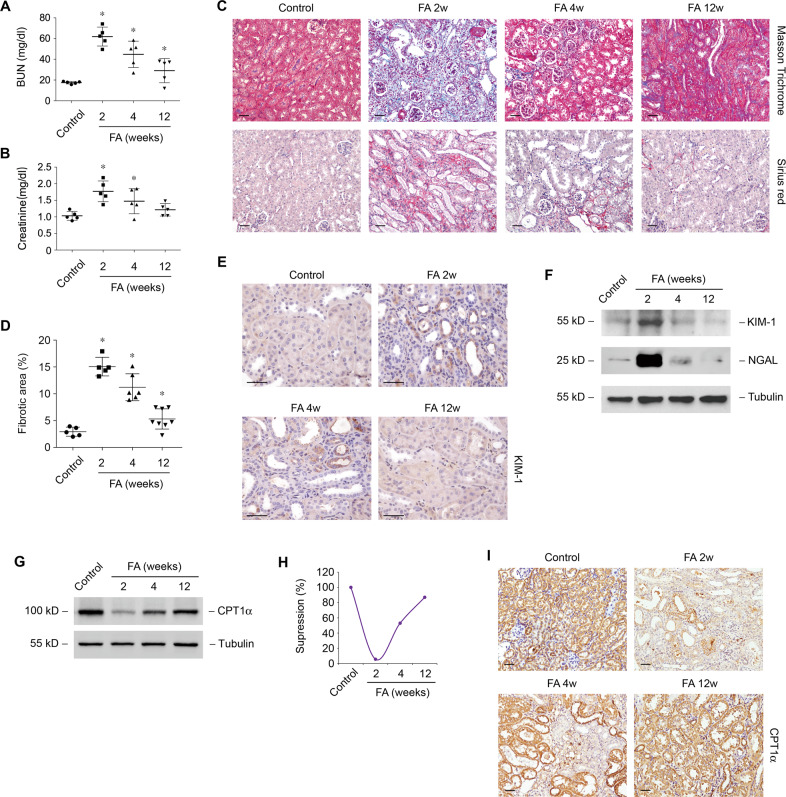
Dynamic changes of CPT1α during kidney injury and repair in folic acid nephropathy model. Changes of BUN (**A**) and creatinine (**B**) in groups as indicated. *n* = 5 for each group. * *P* < 0.05 versus control. **C** Representative images of mice kidney samples from groups as indicated stained with Masson trichrome and Sirius red. **D** Percentage of the fibrotic area in groups as indicated. *n* = 5−8 for each group. * *P* < 0.05 versus control. **E** Representative images of mice kidney samples immunostained with KIM-1. **F** Western blot analysis of protein expression of KIM-1 and NGAL in kidney samples from groups as indicated. **G** Western blot analysis of protein expression of CPT1α in kidney samples from groups as indicated. **H** Graphic presentation of the relative abundance of CPT1α in the folic acid model. **I** Representative images of mice kidney samples immunostained with CPT1α. Scale bar, 50 μm.

Models of kidney injury and repair were established by folic acid nephropathy [19, 26], which was verified by dynamic changes of serum renal function parameters BUN (Fig. 1A) and creatinine (Fig. 1B). Consistently, the morphologic analysis showed that the tubulointerstitial fibrosis induced by folic acid at 2 weeks was relieved spontaneously at 12 weeks (Fig. 1C, D). Tubular epithelial cells injury markers KIM-1 and NGAL barely detected in normal kidney were markedly increased at 2 weeks after folic acid and then declined at 12 weeks by immunohistochemical staining (Fig. 1E) and western blotting (Fig. 1F). Notably, expression of CPT1α was decreased and recovered during kidney injury (at 2 weeks) and repair (from 4 weeks till 12 weeks), respectively (Fig. 1G, H). Immunohistochemical staining showed the dynamic expression of CPT1α in tubular epithelial cells in FA model (Fig. 1I).

### Changes of tubular cell phenotype in FA model

**Fig. 2:**
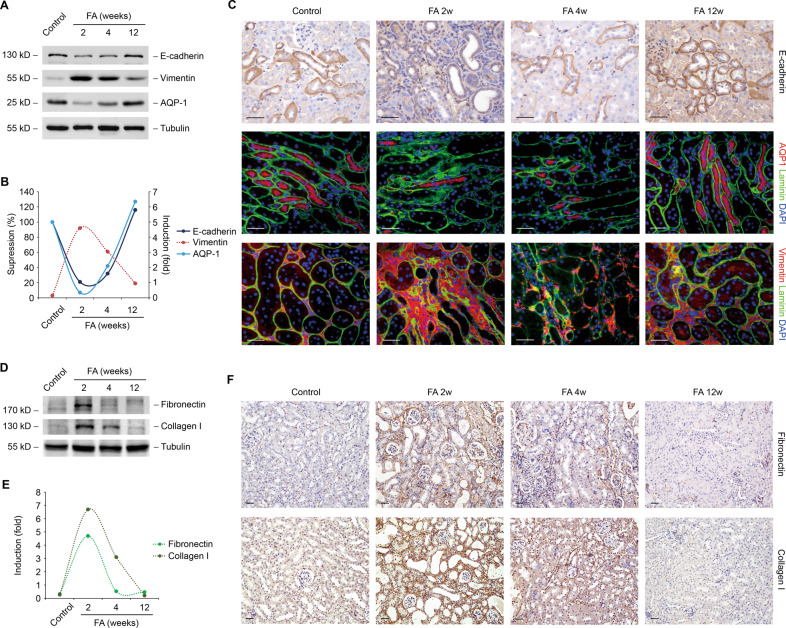
Changes of tubular cell phenotype in FA model. **A** Western blot analysis of protein expression of E-cadherin, vimentin, and AQP-1 in kidney samples from groups as indicated. **B** Graphic presentation of the relative abundance of E-cadherin, vimentin, and AQP-1 in the folic acid model. **C** Representative images of mice kidney samples immunostained with E-cadherin, AQP-1, and vimentin. **D** Western blot analysis of protein expression of fibronectin and collagen I in kidney samples from groups as indicated. **E** Graphic presentation of the relative abundance of fibronectin and collagen I in the folic acid model. **F** Representative images of mice kidney samples immunostained with fibronectin and collagen I. Scale bar, 50 μm.

To gain further insight into the molecular events underlying tubular cell injury and repair, an analysis of tubular cell phenotype from folic acid mice was performed. Several tubular cells markers including E-cadherin and AQP1 were decreased at 2 weeks after folic acid and recovered at 12 weeks by western blotting (Fig. 2A, B) and immunostaining (Fig. 2C). Meanwhile, an opposite alternation was found in the expression of vimentin. Extracellular proteins such as fibronectin and collagen I were upregulated at 2 weeks after folic acid and eliminated at 12 weeks by western blotting (Fig. 2D, E) and immunostaining (Fig. 2F), which were in accordance with the dynamic changes of phenotype markers of tubular cell.

### Genetic ablation of CPT1α aggravates tubular cell injury and fibrosis and hampers kidney repair

**Fig. 3:**
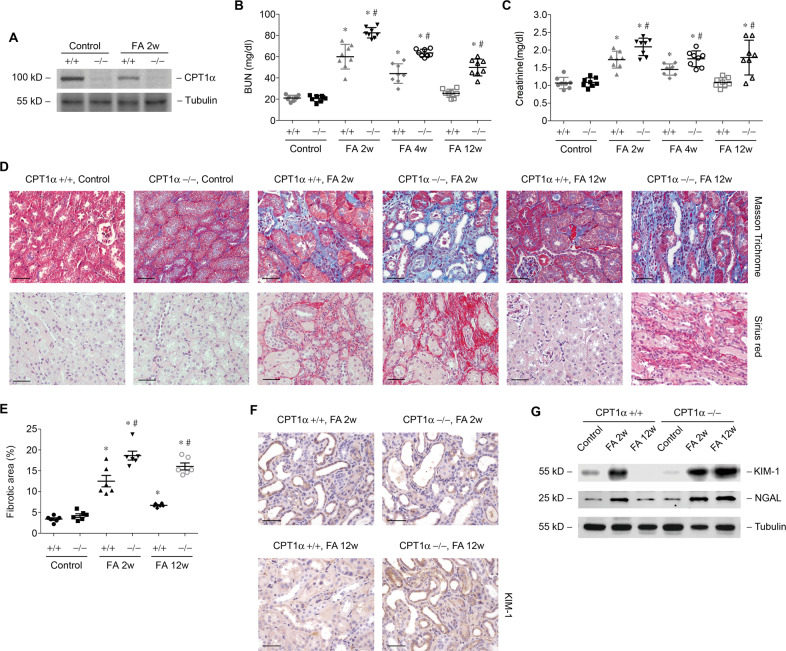
Genetic ablation of CPT1α aggravates tubular cell injury and hampers kidney repair. **A** Western blot analysis of protein expression of CPT1α in kidney samples from groups as indicated. Changes of BUN (**B**) and creatinine (**C**) in groups as indicated. *n* = 8 for each group. **D** Representative images of mice kidney samples from groups as indicated stained with Masson trichrome and Sirius red. **E** Percentage of the fibrotic area in groups as indicated. *n* = 6 for each group. * *P* < 0.05 versus control. # *P* < 0.05 versus CPT1α+/+. **F** Representative images of mice kidney samples immunostained with KIM-1. **G** Western blot analysis of protein expression of KIM-1 and NGAL in kidney samples from groups as indicated. Scale bar, 50 μm.

To further obtain direct evidence that CPT1α within kidney tubule is a direct cause of tubular injury and repair, we generated an inducible, tubular-specific CPT1α knockout mice (CPT1α−/−). Immunoblot analysis of the renal cortex confirmed the ablation of CPT1α in kidney tubule (Fig. 3A). Kidney dysfunction (Fig. 3B, C) and renal fibrosis (Fig. 3D, E) at 2 weeks after folic acid injection were more severe in tubular-specific CPT1α−/− mice. The recovery of kidney function and dissipation of fibrosis were barely absent in CPT1α−/− mice. Genetic ablation of CPT1α resulted in sustained upregulation of KIM-1 and NGAL (Fig. 3F, G) in folic acid mice kidney.

### CPT1α is indispensable for preserve and recovery of tubular phenotype

**Fig. 4:**
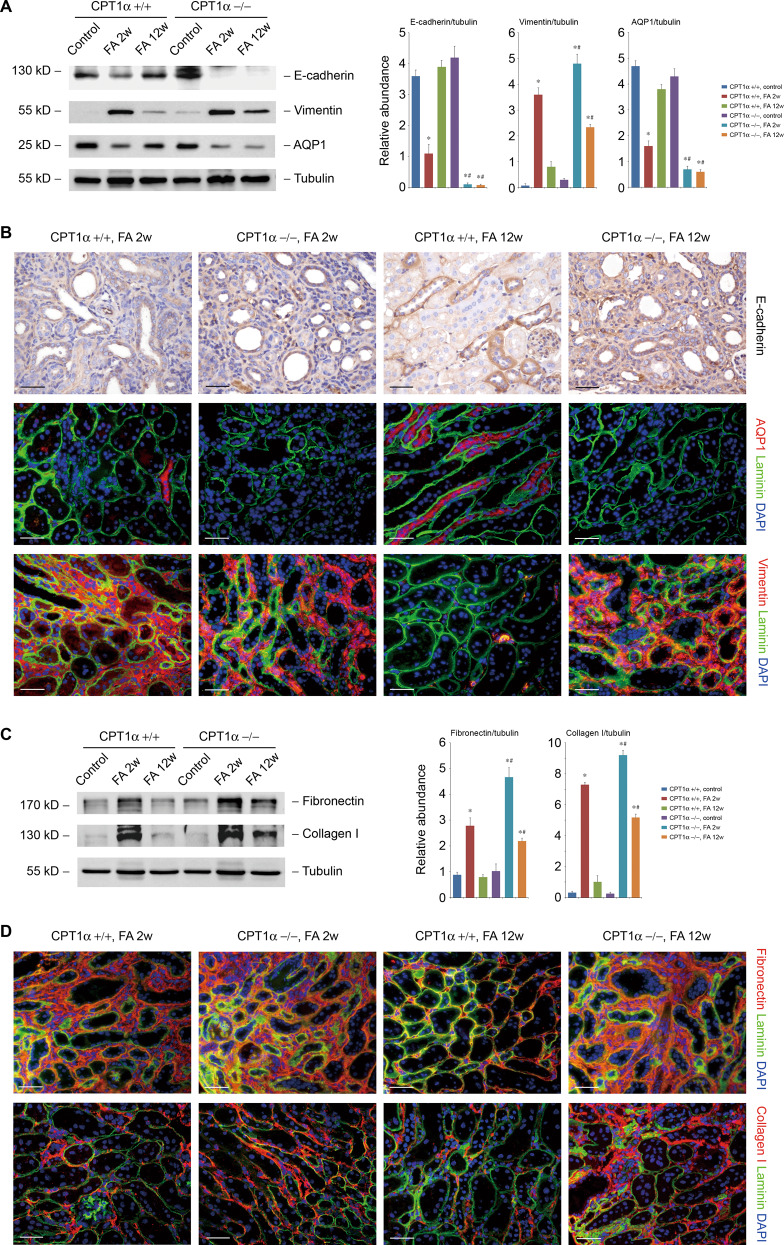
CPT1α is indispensable for preserve and recovery of tubular phenotype. **A** Western blot analysis of protein expression and graphic presentation of the relative abundance of E-cadherin, vimentin, and AQP-1 in kidney samples from groups as indicated. **B** Representative images of mice kidney samples immunostained with E-cadherin, AQP-1, and vimentin. **C** Western blot analysis of protein expression and graphic presentation of the relative abundance of fibronectin and collagen I in kidney samples from groups as indicated. **D** Representative images of mice kidney samples immunostained with fibronectin and collagen I. * *P* < 0.05 versus control. # *P* < 0.05 versus CPT1α+/+. Scale bar, 50 μm.

We further examined the effect of CPT1α deletion on tubular cell phenotype alternation during injury and repair. As compared with CPT1α+/+ mice, the decrease of E-cadherin and AQP1, as well as the increase of vimentin at 2 weeks after folic acid were more significant in CPT1α−/− mice. The altered expression of these phenotype markers persisted at 12 weeks in CPT1α−/− mice by western blotting (Fig. 4A) and immunostaining (Fig. 4B). Similarly, the upregulation (Fig. 4C) and extracellular accumulation (Fig. 4D) of fibronectin and collagen I at 2 weeks after folic acid were more significant in CPT1α−/− mice and do not dissipated at 12 weeks after folic acid.

### CPT1α deficiency promotes lipid accumulation and mitochondrial mass reduction

**Fig. 5:**
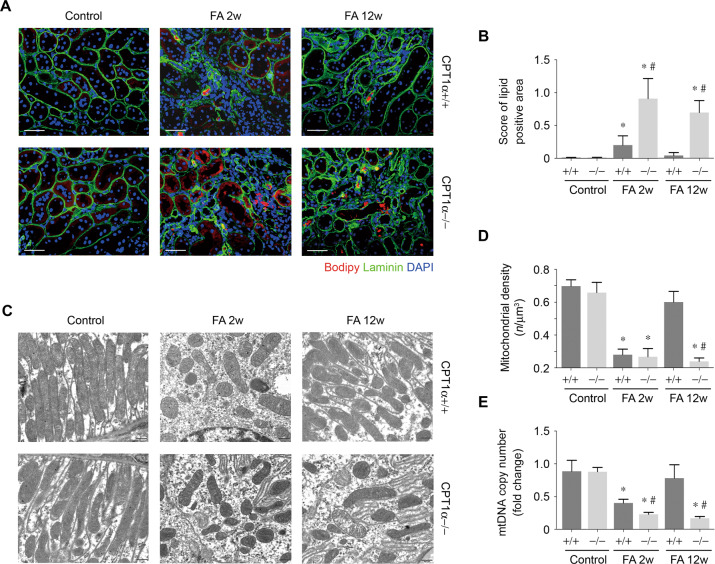
CPT1α deficiency promotes lipid accumulation and mitochondrial mass reduction. **A** Representative images of kidney samples stained with bodipy. Red, bodipy. Green, laminin. Blue, nuclei. Scale bar, 50 μm. **B** Quantification of lipid positive area in kidney samples obtained from groups as indicated. *n* = 6 for each group. **C** Representative TEM of the ultrastructure of mouse kidney tubular cells obtained from resin-embedded kidney sections from groups as indicated. Scale bars: 500 nm. **D** Quantification of mean mitochondria per volume (n/μm^3^) by morphometric analysis. *n* = 6 for each group. **E** mtDNA copy number was determined in kidneys from groups as indicated. Bar graphs represent the mean ± SEM of fold changes. *n* = 6 for each group. * *P* < 0.05 versus control. # *P* < 0.05 versus CPT1α+/+.

There was a higher amount of lipid accumulated within the kidney tubules at 2 weeks after folic acid in CPT1α−/− mice compared to CPT1α+/+ mice, which was exemplified by neutral lipid staining and quantification (Fig. 5A, B). The resolution of lipid in tubule of CPT1α+/+ mice at 12 weeks after folic acid was absent in CPT1α−/− mice. Meanwhile, electron micrographs (Fig. 5C) and quantification using morphometric analysis (Fig. 5D) showed that the number of mitochondria in proximal tubules were markedly reduced at 2 weeks in folic acid mice compared with control. Restoration of mitochondria in CPT1α+/+ mice at 12 weeks after folic acid also did not occur in CPT1α−/− mice. As compared with CPT1α+/+ mice, the decrease of mtDNA copy number (Fig. 5E) after folic acid was more significant at 2 weeks and persistent at 12 weeks in CPT1α−/− mice.

### CPT1α regulates respiration and ATP production in cultured tubular epithelial cells

**Fig. 6:**
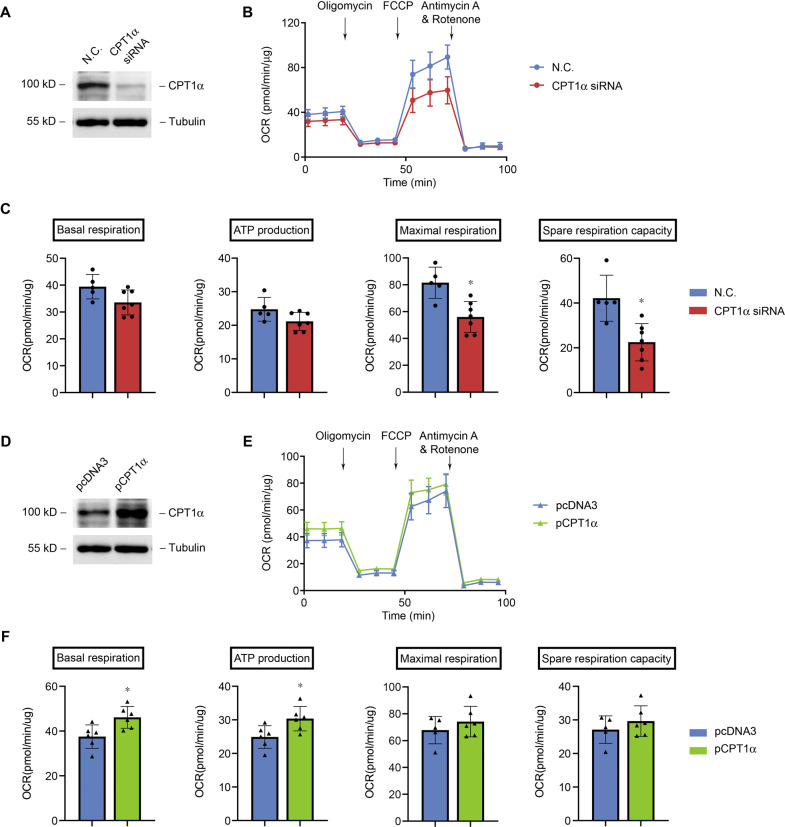
CPT1α regulates respiration and ATP production in cultured tubular epithelial cells. **A** Western blot analysis of protein expression of CPT1α in cultured tubular cells after transfection with N.C. or CPT1α siRNA. **B** Representative traces show OCR in tubular cells. **C** Summary OCR data from 5 to 6 independent experiments are shown. * *P* < 0.05 versus N.C. **D** Western blot analysis of protein expression of CPT1α in cultured tubular cells after transfection with pcDNA3 or CPT1α plasmid. **E** Representative traces show OCR in tubular cells. **F** Summary OCR data analyzed from 5 to 6 independent experiments are shown. * *P* < 0.05 versus pcDNA3. One μmol/L Oligomycin, 0.75 μmol/L of FCCP, 1 μmol/L of antimycin A and rotenone were added where indicated.

We modulated the expression of CPT1α in primarily cultured tubular epithelial cells by transfection of CPT1α siRNA or plasmid. Immunoblot revealed a markedly decrease in CPT1α protein expression after specific siRNA transfection compared to negative control (N.C.) siRNA (Fig. 6A). Tubular cells with less CPT1α had reduced baseline OCR and a lower ATP production. The FCCP-induced maximal respiration and spare respiration capacity were markedly reduced after the downregulation of CPT1α (Fig. 6B, C). On the contrary, specific plasmid transfection induced a markedly increase in CPT1α protein expression compared to the control plasmid (pcDNA3) (Fig. 6D). In tubular cells with upregulated CPT1α, the baseline OCR and ATP production were elevated remarkably. The FCCP-induced maximal respiration and spare respiration capacity also showed an upward trend after the upregulation of CPT1α (Fig. 6E, F). These results indicate that CPT1α promotes the activity of mitochondrial respiration.

### Tubular cells with reduced CPT1α are more sensitive to folic acid-induced alternation

**Fig. 7:**
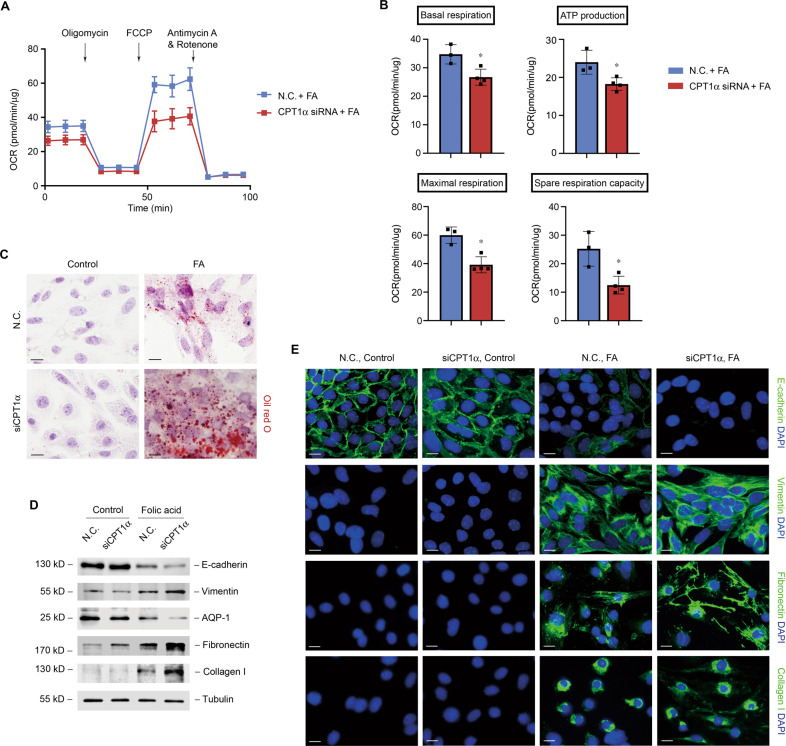
Tubular cells with reduced CPT1α are more sensitive to folic acid-induced alternation. **A** Representative traces show OCR in folic acid-treated tubular cells after transfection with N.C. or CPT1α siRNA. Oligomycin, FCCP, antimycin A, and rotenone were added where indicated. **B** Summary OCR data from 3 to 4 independent experiments are shown. * *P* < 0.05 versus N.C. + FA. **C** Representative images of tubular cells exposed to treatments as indicated stained with oil red O. **D** Western blot analysis of protein expression of E-cadherin, vimentin, AQP-1, fibronectin, and collagen I in tubular cells. **E** Representative images of tubular cells immunostained with E-cadherin, vimentin, fibronectin, and collagen I. Scale bar, 10 μm.

We next provided specific evidence by measuring the effect of CPT1α siRNA on lipid accumulation and tubular cell phenotype in folic acid treatment conditions. Knockdown of CPT1α markedly aggravated the reduction of baseline OCR, maximal respiration, spare respiration capacity, and ATP production (Fig. 7A, B) and resulted in more lipid accumulation (Fig. 7C). Folic acid-induced suppression of tubular markers (E-cadherin and AQP1) and promotion of vimentin and ECM markers (fibronectin and collagen I) were more remarkable by the interference of CPT1α compared to control (Fig. 7D, E).

### CPT1α protects mitochondrial respiration and prevents against folic acid-induced tubular cell damage

**Fig. 8:**
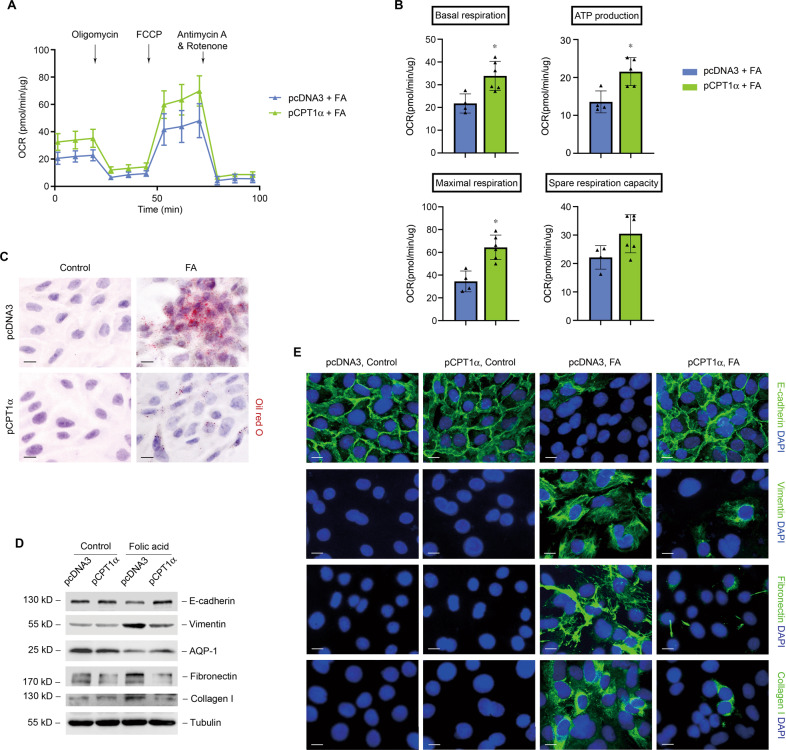
CPT1α protects mitochondrial respiration and prevents against folic acid-induced tubular cell damage. **A** Representative traces show OCR in folic acid-treated tubular cells after transfection with pcDNA3 or CPT1α plasmid. Oligomycin, FCCP, antimycin A, and rotenone were added. **B** Summary OCR data from 3 to 4 independent experiments are shown. * *P* < 0.05 versus pcDNA3 + FA. **C** Representative images of tubular cells exposed to treatments as indicated stained with oil red O. **D** Western blot analysis of protein expression of E-cadherin, vimentin, AQP-1, fibronectin, and collagen I in tubular cells. **E** Representative images of tubular cells immunostained with E-cadherin, vimentin, fibronectin, and collagen I. Scale bar, 10 μm.

We next measured the effect of overexpression of CPT1α on lipid accumulation and tubular cell phenotype in folic acid treatment conditions. Overexpression of CPT1α by transfection of specific plasmid markedly alleviated the reduction of baseline OCR, maximal respiration, spare respiration capacity, and ATP production (Fig. 8A, B) and resulted in reduced lipid accumulation (Fig. 8C). Folic acid-induced suppression of tubular markers (E-cadherin and AQP1) and promotion of vimentin and ECM markers (fibronectin and collagen I) were markedly relieved by upregulation of CPT1α compared to control (Fig. 8D, E).

## Discussion

In this study, we have identified CPT1α as a central molecule involved in the injury and repair response of damaged renal tubule cells. In vivo, tubular-specific ablation of CPT1α leads to sustained tubular damage and absence of the normal recovery of kidney function and dissipation of fibrosis after folic acid injury. In vitro, CPT1α promotes mitochondrial respiration and ATP production in cultured tubular epithelial cells. Moreover, we demonstrate that CPT1α relieves kidney injury and promotes tubular repair by preservation and recovery of tubular phenotype. Our results highlight the metabolism of proximal tubule cellular as one of the major drivers of cellular dedifferentiation and redifferentiation during kidney injury and repair.

The harmoniously coupling of energy metabolism and cellular state makes perfect sense in physiological conditions, especially in the energy-consuming proximal tubule. High glycolysis in effector T cells, while higher FAO exhibited in regulatory cells were the best-described example [27]. Dysregulated metabolism including increased glycolysis, OXPHOS, and fatty acid synthesis contributes to autoimmune diseases [28–30]. The remarkable effect of sodium-glucose cotransporter 2 inhibitors in relieving the deterioration of renal function may possibly attribute to modulating tubular energy metabolism [31, 32]. It is recently reported that proximal tubular cell differentiation is altered in kidney disease, which shows the strongest and most reproducible association with metabolism, especially FAO and OXPHOS [14]. In this study, we established the role of CPT1α, the rate-limiting enzyme of FAO in the coupling of tubular cell phenotype and the metabolism.

CPT1 enzyme has three isoforms with tissue-specific expression and encoded by a different gene. The liver and kidney CPT1α is expressed by CPT1A gene, while the skeletal and cardiac muscle by the CPT1B gene, and the brain by the CPT1C gene [33]. Although increasing evidence has demonstrated an association between the decreased expression of CPT1α and kidney diseases development [6], more direct evidence is still lacking. We, therefore, breed tetO-CPT1α mice with Pax8rt-TA mice and feed them with a doxycycline-containing diet to generate the inducible tubular-specific CPT1α deletion in adult mice and therefore avoid the embryo and pre-mature lethality. CPT1 deficiency in clinical settings is an autosomal-recessively inherited condition mainly affecting liver, heart, muscle, and kidney. Half a century ago, a case of CPT1 deficiency was reported with clinical manifestation of kidney involvement, including pigmenturia and rhabdomyolysis [34]. However, the symptoms are intermittent and induced by certain stress, and the stimulus that triggers clinical presentations differ between each patient [35, 36]. Not surprisingly, we found no significant difference in the phenotype of knockout (CPT1α−/−) and their littermates (CPT1α+/+) at the time before the folic acid injury. Although the basal OCR tends to decreased in CPT1α deficient PTECs, the tubular phenotype and lipid remain normal. Whether there is a compensatory mechanism for FAO in the absence of CPT1α in normal conditions needs further investigations.

Of note, the severity of acute kidney injury induced by folic acid was comparable between CPT1α+/+ and CPT1α−/− mice (data not shown). It was reported that metabolic switch from FAO toward glycolysis that occurred early after acute injury was indispensable for tubular regeneration and recovery [9]. This even coincides with a previous study suggesting inhibition of the CPT enzyme to protect PTECs during hypoxia [37]. We postulated that PTECs may become less dependent on FAO in the acute phase after injury. However, the irreversibility of the metabolic switch characterizes persistent dedifferentiation of PTECs and results in severe renal fibrosis in the late phase [9].

As the rate-limiting enzyme for medium and long fatty acid shuttling into mitochondria, ablation of CPT1α certainly hampered the recovery of preferred FAO in PTECs and results in more severe fatty acid deposition and tubulointerstitial fibrosis. Verónica Miguel found a correlation between short-/medium-acylcarnitine levels and renal function [18]. Afshinnia et al. found impaired fatty acid β-oxidation in severe kidney disease, but they did not check the CPT1α levels [38]. In patients with diabetes, kidney involvement is associated with incomplete FAO and alternations of related enzymes including CPT1α [6]. Here, the recovery of FAO, accompanied with CPT1 most likely contributes to kidney repair; however, as CPT1α is rate-limiting for FAO in the mitochondria, and overexpression of CPT1α mitigate FAO impairment [18], a clear distinction between the roles of CPT1α and FAO in kidney injury and repair remains obscure.

Mitochondria are the most important intracellular organelles for ATP production. The active reabsorption of large quantities of solutes in kidney tubule needs high energy that relies on mitochondrial oxidation. As the greatest reabsorption section, the proximal tubules primarily apply aerobic respiration for ATP production [2] and equipped with abundant of mitochondria. Quality control and homeostasis of mitochondria are indispensable for the maintenance of a variety of cellular processes in normal kidney. It is conceivable that mitochondria are closely associated with kidney injury and repair. After the initial insult, mitochondrial mass reduction together with the metabolic transfer from OXPHOS to anaerobic glycolysis observed in kidney tubules led to dedifferentiation and proliferation of tubular cells [9]. Reversal of the reduction promotes normal repairment of tubules. However, persist and progressive damage results in failure of tubular redifferentiation, suggesting a key role of mitochondrial regression during the repair. A recent study has suggested that overexpression of CPT1α protects from kidney fibrosis by restoring mitochondrial homeostasis [18]. Mitochondria regulate cell differentiation mainly dependent on the control of energy metabolism. Surviving tubular cells undergo a series of continuous alternations to repair injured kidney tubules, including dedifferentiation, proliferation, migration, and finally redifferentiation into mature tubular cells. Increasing evidence suggests that mitochondrial dysfunction contributes critically to the pathogenesis of injury and incomplete kidney repair. Mitochondrial protection before the injury is protective [39], while mitochondrial protection after injury mitigates the progression to tubular atrophy and chronic fibrosis [40].

In summary, we showed the vital role of metabolism in driving PTECs phenotype state. CPT1α couples cell metabolism and differentiation state by regulating mitochondrial respiration. The work provides new opportunities to manipulate proximal tubular cell differentiation, metabolism, and kidney tissue fate based on the reliance of tubular function on energy metabolic molecules such as CPT1α.
